# Human Natural Antibodies to Mammalian Carbohydrate Antigens as Unsung Heroes Protecting against Past, Present, and Future Viral Infections

**DOI:** 10.3390/antib9020025

**Published:** 2020-06-22

**Authors:** Uri Galili

**Affiliations:** Department of Medicine, Rush University Medical School, Chicago, IL 60605, USA; uri_galili@rush.edu or uri.galili@rcn.com; Tel.: +1-312-753-5997

**Keywords:** anti-Gal, anti-Neu5Gc, *α*-gal epitope, anti-Forssman antibody, Neu5Gc, blood group Bombay

## Abstract

Human natural antibodies to mammalian carbohydrate antigens (MCA) bind to carbohydrate-antigens synthesized in other mammalian species and protect against zoonotic virus infections. Three such anti-MCA antibodies are: (1) anti-Gal, also produced in Old-World monkeys and apes, binds to *α*-gal epitopes synthesized in non-primate mammals, lemurs, and New-World monkeys; (2) anti-Neu5Gc binds to Neu5Gc (*N*-glycolyl-neuraminic acid) synthesized in apes, Old-World monkeys, and many non-primate mammals; and (3) anti-Forssman binds to Forssman-antigen synthesized in various mammals. Anti-viral protection by anti-MCA antibodies is feasible because carbohydrate chains of virus envelopes are synthesized by host glycosylation machinery and thus are similar to those of their mammalian hosts. Analysis of MCA glycosyltransferase genes suggests that anti-Gal appeared in ancestral Old-World primates following catastrophic selection processes in which parental populations synthesizing *α*-gal epitopes were eliminated in enveloped virus epidemics. However, few mutated offspring in which the *α*1,3galactosyltransferase gene was accidentally inactivated produced natural anti-Gal that destroyed viruses presenting *α*-gal epitopes, thereby preventing extinction of mutated offspring. Similarly, few mutated hominin offspring that ceased to synthesize Neu5Gc produced anti-Neu5Gc, which destroyed viruses presenting Neu5Gc synthesized in parental hominin populations. A present-day example for few humans having mutations that prevent synthesis of a common carbohydrate antigen (produced in >99.99% of humans) is blood-group Bombay individuals with mutations inactivating H-transferase; thus, they cannot synthesize blood-group O (H-antigen) but produce anti-H antibody. Anti-MCA antibodies prevented past extinctions mediated by enveloped virus epidemics, presently protect against zoonotic-viruses, and may protect in future epidemics. Travelers to regions with endemic zoonotic viruses may benefit from vaccinations elevating protective anti-MCA antibody titers.

## 1. Introduction

Natural antibodies in humans are continuously produced without active vaccination. The much higher susceptibility to viral and bacterial infections of mice lacking immunoglobulins compared to mice producing natural antibodies led to the suggestion that “Natural antibodies are often dismissed from immunological analysis as ‘background’, but they may play an important role in conferring immunity against infections” [1]. A large proportion of the natural antibodies in humans bind to various carbohydrate-antigens [2,3,4]. Many natural anti-carbohydrate antibodies are produced as a result of antigenic stimulation by multiple carbohydrate-antigens on gastrointestinal (GI) bacterial flora [5,6]. It is estimated that humans harbor nearly 10^14^ intestinal bacteria [7] of ~400 different strains [8], all covered with multiple immunogenic carbohydrate-antigens. Studies using recently developed carbohydrate microarrays technology have demonstrated in human serum natural anti-carbohydrate antibodies binding to >100 different carbohydrate-antigens [2,3,4]. The most known human natural anti-carbohydrate antibodies are those produced against blood-groups A and B of the ABO system, also produced against GI bacterial carbohydrate-antigens [9]. This review describes a group of human natural anti-carbohydrate antibodies of particular significance, which may be regarded as “unsung heroes” that function as an important first line of immune defense in humans against zoonotic enveloped viruses. Although they are produced against bacterial carbohydrate-antigens, these natural antibodies also bind to mammalian carbohydrate-antigens (MCA) produced in species other than humans.

The protective effects of natural anti-MCA antibodies are associated with a basic biosynthetic principle, common to enveloped viruses: these viruses present carbohydrate-antigens, synthesized on their envelope glycoproteins by the host-cell glycosylation machinery. Therefore, the carbohydrate-antigens on enveloped viruses act as an immunologic “Achilles Heel”, which enable destruction of deleterious viruses by natural anti-MCA antibodies. This review describes three of the most studied anti-MCA antibodies, readily detected in humans (Table 1), namely anti-Gal, anti-N-glycolyl neuraminic acid (anti-Neu5Gc), and anti-Forssman antibodies, all found in healthy individuals. Based on the mammalian distribution patterns of these antibodies, on their corresponding MCA ligands, and on the characteristics of the genes coding enzymes that synthesize these antigens, this review further suggests that natural anti-MCA antibodies protected ancestral primates from extinction in past epidemics of deadly enveloped viruses and presently continue such a protection.

A fourth antibody described in this review is the anti-blood-group O (anti-H) antibody, which is naturally produced in only <0.001% of humans. These are blood-group Bombay individuals. Anti-H serves as a present-day example which simulates the potential of anti-MCA antibodies in preventing extinctions of few mutated primates during deadly viral epidemics and may have a similar role in future epidemics.

## 2. Anti-MCA Antibodies

The characteristics of the three human anti-MCA antibodies listed in Table 1 are as follows.

### 2.1. Anti-Gal Antibody

**Figure 1:**
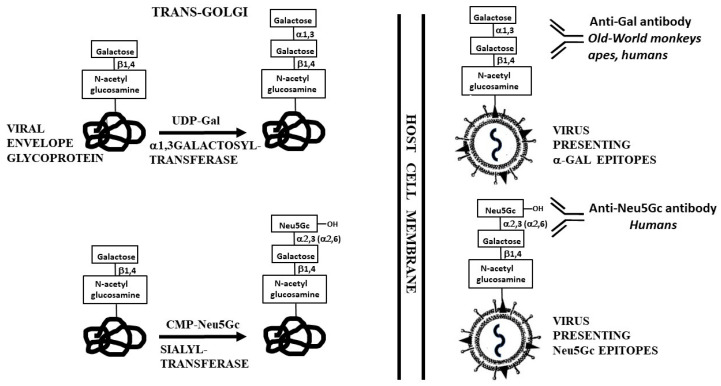
Synthesis of *α*-gal epitopes and Neu5Gc on enveloped virus glycoproteins in the trans-Golgi compartment of host-cells. The synthesis is mediated by *α*1,3galactosyltransferase and sialyltransferase, using UDP-Gal and CMP-Neu5Gc as sugar donors, respectively. The hydroxyl of Neu5Gc is illustrated as well. From the transGolgi, the glycoprotein molecules are transported to the cell membrane where they assemble into the virus envelope, followed by budding of the virus particles from the infected cells. The antibodies binding to these MCA and the species producing them are indicated.

Anti-Gal is an abundant antibody in human constituting ~1% of serum IgG, IgM, and IgA immunoglobulins [10,11,12,13] and is found as IgA and IgG in body secretions including milk, colostrum, saliva, and bile [11]. Approximately 1% of human blood B cells can produce anti-Gal [14], most of which are quiescent and produce the antibody upon activation. Anti-Gal is produced throughout life [15] as a result of antigenic stimulation by carbohydrate-antigens on GI bacteria [16,17,18]. The MCA binding anti-Gal is called the *α*-gal epitope, with the structure Gal*α*1-3Gal*β*1-4GlcNAc-R on carbohydrate chains (collectively called “glycans”) linked to mammalian glycoproteins, glycolipids, and proteoglycans [19,20,21] (Figure 1). Anti-Gal is a polyclonal antibody and its heavy chain is mostly coded by IgH genes clustered in the VH3 family [22]. In blood-group A and O individuals, anti-Gal also comprises >80% of the anti-blood-group B activity. This is indicated by the binding of a large proportion of serum anti-blood group B antibodies both to the *α*-gal epitope (Gal*α*1-3Gal*β*1-4GlcNAc-R) and to blood-group B antigen (Gal*α*1-3(Fuc*α*1-2)Gal*β*1-4GlcNAc-R), which has a structure identical to the *α*-gal epitope and an additional fucose linked *α*1-2 to the penultimate galactose [23]. The *α*-gal epitope is found as ~10^5^–10^7^ epitopes/cell in all mammals that are not monkeys or apes (non-primate mammals), in lemurs (prosimians that evolved in Madagascar), and in monkeys of South America (New-World monkeys), but is absent in non-mammalian vertebrates [24,25]. The *α*-gal epitope is synthesized by the enzyme *α*1,3galactosyltransferase (*α*1,3GT) [25,26]. Mammals synthesizing *α*-gal epitopes do not produce anti-Gal because of immune tolerance to this self-antigen [24]. In contrast, Old-World monkeys (monkeys of Asia and Africa), apes, and humans completely lack *α*-gal epitopes due to evolutionary inactivation of the *α*1,3galactosyltransferase gene (also called *α1,3GT* gene, or *GGTA1*) [24,25,27,28,29,30], and all produce the natural anti-Gal antibody [10,24,31]. Anti-Gal readily binds in vivo to *α*-gal epitopes and activates the complement system as a result of this interaction. This is demonstrated by the rapid (hyperacute) rejection of porcine cells and organs (i.e., xenografts) transplanted into Old-World monkeys [32,33,34,35,36,37].

### 2.2. Anti-Neu5Gc Antibody

The carbohydrate antigen *N*-5-glycolyl-neuraminic acid (Neu5Gc) is produced by hydroxylation of *N*-acetyl-5-neuraminic acid (Neu5Ac) linked to cytosine-monophosphate (CMP), forming CMP-Neu5Ac-OH, which is CMP-Neu5Gc [38]. Both terminal Neu5Ac and Neu5Gc are found on glycans in most non-primate mammals as well as in Old-World monkeys and apes [39,40,41]. Humans lack Neu5Gc and synthesize only Neu5Ac, because of inactivation of the gene coding the enzyme cytidine-monophosphate-*N*-acetyl-neuraminic acid hydroxylase (*CMAH*). This inactivation is due to a 92-bp deletion, which generates a frameshift mutation that forms a pre-mature stop codon [42,43]. Instead, humans produce the natural anti-Neu5Gc antibody, originally called Hanganutziu–Deicher antibody [44,45,46,47], which specifically interacts with Neu5Gc linked to glycans on cells. Thus, the mere addition of a hydroxyl to Neu5Ac (thereby converting it to Neu5Gc) generates a xenoantigen specifically binding the human natural anti-Neu5Gc antibody (Figure 1). Similar to anti-Gal, the natural anti-Neu5Gc antibody binds in vivo to cells presenting Neu5Gc, mediates their complement dependent cytolysis and thereby may contribute to xenograft rejection [45,46,47,48,49].

### 2.3. Anti-Forssman Antibody

This antibody was identified as a natural antibody in human serum by its binding to sheep red blood cells (RBC) and causing complement dependent cytolysis of these RBC [50,51]. Therefore, it has been referred to also as “heterophil” antibody and it is produced in humans as IgM, IgA, and IgG antibodies. Anti-Forssman binds to Forssman-antigen (named after the Swedish pathologist J.F. Forssman) with the structure *N*-acetyl galactosamine (GalNAc) linked *α*1-3 to a penultimate GalNAc (GalNAc*α*1-3GalNAc-R) on glycolipids [52,53]. The gene coding the glycosyltransferase synthesizing the Forssman-antigen (*GBGT1*) displays sequence homology with blood-group A and B transferase and with *α1,3GT* (*GGTA1*) genes [54]. Whereas the Forssman-antigen is abundant in tissues of chicken [55], sheep, horse, dog, cat, mouse, hamster, and guinea-pig, it is absent in humans, rat, cow, pig, and rabbit [56]. In humans, *GBGT1* is a pseudogene because of two missense mutations [57]. Instead, humans produce the natural anti-Forssman antibody, which activates the complement system following its interaction with the Forssman-antigen. In a recent study, anti-Forssman antibody production was reported in sera of 799 out of 800 individuals tested [58].

## 3. Biosynthesis of MCA on Enveloped Viruses

Glycans found on cell membranes are synthesized in the endoplasmic reticulum and subsequently in the Golgi apparatus in a sequential process analogous to assembly lines in car plants. Glycosyltransferases add to the nascent carbohydrate chain various carbohydrate units provided by high energy sugar donors such as UDP-Gal and CMP-Neu5Gc [59]. Enveloped viruses lack genes coding glycosyltransferases and use the host-cell glycosylation machinery to synthesize glycans on their envelop glycoproteins. A schematic illustration of the last step (also called “capping”) in the synthesis of *α*-gal and Neu5Gc epitopes is presented in Figure 1. Thus, the carbohydrate-antigen profile on enveloped viruses is dependent on the host-cell. Accordingly, Eastern-equine-encephalitis (EEE) virus produced in mouse 3T3 fibroblasts presents *α*-gal epitopes, whereas the same virus produced in Vero cells (African green monkey—Old-World monkey) completely lacks this epitope [60]. Similarly, influenza virus produced in bovine or canine cells presents *α*-gal epitopes, but when produced in chicken cells (cells lacking *α*1,3GT) it lacks these epitopes [61]., Vesicular stomatitis virus (VSV) glycoproteins produced in human cells lack *α*-gal epitopes but carry this epitope if the human host-cells were transfected with the *α1,3GT* gene [62]. Similarly, VSV produced in human cells lacks Neu5Gc antigen, whereas VSV produced in Vero cells presents Neu5Gc on its envelope glycoproteins [62]. The ultimate number of *α*-gal epitopes per virion may differ from one type of virus to the other and depends both on the number of glycans on the virus envelope and on the competition between *α*1,3GT and other glycosyltransferases (e.g., sialyltransferase) for “capping” glycans with terminal *α*1-3Gal or with other carbohydrate units within the trans-Golgi compartment [63,64]. In-vitro synthesis of *α*-gal epitopes on influenza virus by recombinant *α*1,3GT and UDP-Gal, results in expression of as many as 3000 *α*-gal epitopes per virion [65]. However, the actual number of these epitopes on virions produced in host-cells is likely to be lower because of the competition with other glycosyltransferases [63,64].

## 4. Anti-Gal Antibody Protection against Zoonosis

All humans who are not severely immunocompromised produce anti-Gal, which is likely to provide some protection against viruses carrying *α*-gal epitopes. Several observations support this assumption. Incubation of EEE virus presenting *α*-gal epitopes (i.e., virus produced in mouse cells) with anti-Gal IgG purified from human serum, had a significant neutralizing effect on the virus, indicated by the subsequent 50% decrease in plaque formation in infected Vero cells, whereas no neutralization was detected with EEE virus lacking *α*-gal epitopes, which was produced in Vero cells [60]. However, much more effective neutralization and killing (i.e., virolysis) effects of anti-Gal were observed with viruses incubated in human serum containing both anti-Gal and complement. In-vitro complement mediated virus killing by anti-Gal binding to *α*-gal epitopes on viral envelope glycoproteins was observed upon incubation in human serum of several types of enveloped viruses, including: Type C retrovirus [66], porcine endogenous retrovirus (PERV) [67,68], vesicular stomatitis virus (VSV) [62,69,70], lymphocytic choriomeningitis virus, Newcastle disease virus, Sindbis virus [70], Pseudorabies virus (PrV—a member of herpesviridae *α*-herpes subfamily) [71], measles virus [72,73], and vaccinia virus [74], all produced in host-cells with active *α*1,3GT. Such incubation was found to kill as many as 99.99% of VSV particles, whereas in the absence of *α*-gal epitopes on the virus, neutralization and killing upon incubation in human serum was ~100-fold less potent [62]. All these observations on anti-Gal mediated neutralization and destruction of viruses have suggested that anti-Gal may serve as a barrier against infections by zoonotic viruses presenting *α*-gal epitopes.

It is impossible to determine the extent of anti-Gal mediated protection in humans against zoonotic viruses carrying *α*-gal epitopes since there are no control populations lacking this antibody. The potency of anti-Gal as a natural anti-MCA antibody protecting against viral infections may be inferred from an analogous scenario of the Severe acute respiratory syndrome (SARS) corona virus (SARS-CoV) from a single SARS patient infecting hospital workers in Hong Kong, in the SARS outbreak of 2002–2003 [75]. Most of the infected workers were of blood-groups A, B, and AB, whereas blood-group O individuals were relatively resistant to this infection. In-vitro studies demonstrated anti-blood-group A antibodies binding to blood-group A on SARS-CoV and inhibiting infection of cells by the virus [76]. These observations suggested that blood-group O individuals are more resistant to SARS-CoV due to anti-blood group A and anti-blood group B (ABO) antibodies that decrease the rate of infection throughout the population [76]. The proportion of anti-Gal producing B cells is 4–5-fold higher than that of B cells producing ABO antibodies, suggesting a higher protective anti-Gal activity even than that of ABO antibodies [14]. Taken together, the observations above on anti-Gal mediated destruction of viruses presenting *α*-gal epitopes and the protective effect of ABO antibodies against SARS-CoV in humans, strongly suggest that anti-Gal can indeed function in humans as a barrier to zoonotic infections by enveloped viruses produced in hosts synthesizing *α*-gal epitopes. Evidently, this anti-Gal mediated protection may not be absolute and is likely to be associated with the number of *α*-gal epitopes on different viruses, activity of anti-Gal in various individuals and the virulence of each virus. The annual outbreak of influenza infections originating in porcine reservoirs suggests that anti-Gal is not sufficiently effective in preventing infections by this virus. In contrast, the complete absence of any indication that PERV infects humans exposed to pig tissue implants (e.g., pig skin used for treating patients with burns) [77] may reflect an effective anti-Gal barrier against this virus.

## 5. Anti-Gal Amplifies Immune Response to Viruses with *α*-Gal Epitopes

In addition to direct neutralization/killing of zoonotic viruses, anti-Gal may greatly amplify the anti-viral specific immune response by effectively targeting of the killed virus and its glycoproteins to antigen presenting cells (APC). Immunogenicity of viral vaccines increases when the vaccine is immunocomplexed with the corresponding antibody, resulting in effective targeting to APC due to interactions between the Fc “tail” of the immunocomplexed antibody and Fc receptors on APC. This further results in increased processing and cross-presentation of vaccinating antigens by the APC [78,79,80]. Similarly, immune complexes formed by human anti-Gal binding to *α*-gal epitopes on zoonotic viruses are likely to amplify the protective immune response against specific virus protein antigens by targeting the virus for extensive uptake into APC, via Fc/Fc receptors interaction [81]. This amplified immune response may slow the progression of infections by zoonotic viruses, which “lose” the *α*-gal epitopes. Such loss of the *α*-gal epitope occurs when a small proportion of the infectious zoonotic viruses “succeed” to evade the anti-Gal barrier and replicate within infected human cells (e.g., infection of the cells lining the airways, which, as with all human cells, lack *α*1,3GT).

**Figure 2:**
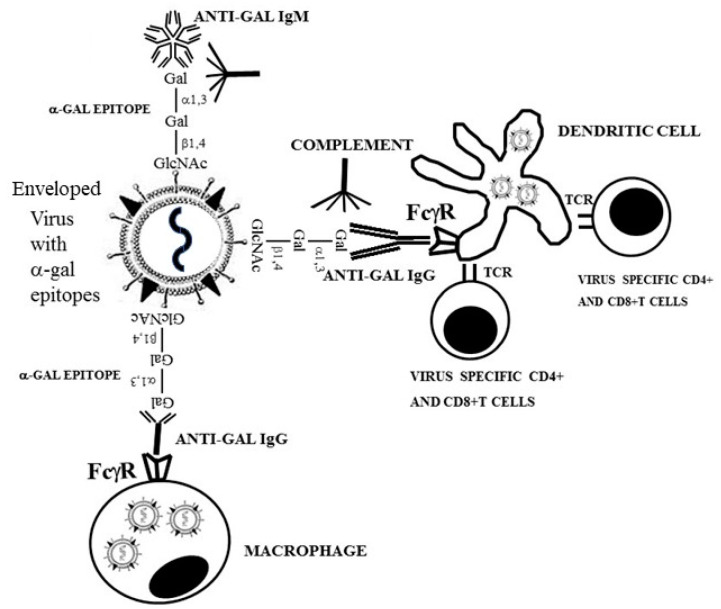
Anti-Gal mediated targeting of virus to antigen presenting cells (APC), such as dendritic cells and macrophages, amplifies the anti-virus specific protective immune response in humans. Binding of anti-Gal to *α*-gal epitopes on enveloped virus glycoproteins induces destruction and neutralization of the virus. The Fc portion of anti-Gal IgG coating the virus further binds to Fcγ receptors (FcγR) on the APC, thereby inducing effective uptake of the antibody coated virus and virus glycoproteins immunocomplexed with anti-Gal, into the APC. These APC transport the internalized virus and viral glycoproteins to lymph nodes, process and present viral immunogenic peptides. The immunogenic peptides presented on major histocompatibility complex (MHC) molecules of APC activate virus specific CD8+ and CD4+ T cells, thereby inducing effective cellular and antibody immune responses against the infecting virus. TCR- T cell receptor.

Increased processing and presentation of immunogenic viral peptides following in-vitro formation of anti-Gal/virus immune complexes was demonstrated by incubation of influenza virus [61] and measles virus [73] presenting *α*-gal epitopes in cultures containing anti-Gal, virus specific T cells, and APC. Such incubation resulted in a much higher proliferation of virus specific T cells than similar incubation with virus lacking *α*-gal epitopes. This increased targeting of anti-Gal coated virus to APC result in increased processing and cross-presentation of viral antigens by APC (Figure 2). Accordingly, in-vivo studies with *α*1,3GT knockout mice (GT-KO mice) producing anti-Gal indicated that immunization with 1 μg inactivated influenza virus presenting *α*-gal epitopes and adjuvant produced anti-influenza virus antibodies at titers that were ~100-fold higher than those produced in mice immunized with a similar dose of influenza virus lacking the *α*-gal epitope and administered with adjuvant [82]. The number of virus specific CD4^+^ T cells also was found to be >100-fold higher and that of specific CD8^+^ T cells six-fold higher, in anti-Gal producing mice immunized with inactivated virus presenting *α*-gal epitopes than in mice immunized with virus lacking the epitope, as measured by intracellular cytokine staining for IFNγ production. Moreover, intranasal challenge studies with a lethal dose of live influenza virus lacking *α*-gal epitopes demonstrated eight-fold higher survival of mice in the former than in the latter group [82].

A similar increased in-vivo immunogenicity of viral glycoprotein presenting *α*-gal epitopes was demonstrated with a 5-μg vaccine of recombinant gp120 of HIV. This amount of vaccinating gp120 lacking *α*-gal epitopes did not elicit a significant anti-gp120 immune response [83,84]. A marked elevation of immune response to a glycoprotein carrying *α*-gal epitopes was also observed in anti-Gal producing GT-KO mice immunized with bovine albumin coupled with synthetic *α*-gal epitopes, in comparison to mice immunized with bovine albumin lacking *α*-gal epitopes [85]. These observations suggest that anti-Gal mediated targeting to APC of even low amounts of zoonotic viruses presenting *α*-gal epitopes, may contribute to the activation of the immune system to react effectively against de-novo produced virions (lacking *α*-gal epitopes) and against cells infected with the virus. This immune response may slow the progression of the infection and enable the protective anti-virus immune response to “catch up” with the infecting virus before the replicating virus reaches lethal stages of the infection. In-vivo effective targeting of vaccines to APC by anti-Gal/*α*-gal epitopes interaction was further found to elicit a protective immune response against distant metastases, by intratumoral injection of *α*-gal glycolipids into solid tumor. This procedure induces expression of *α*-gal epitopes on the tumor cells and converts these cells into an in-situ autologous tumor vaccine targeted for extensive uptake by APC [86].

## 6. Increasing Anti-Gal Titers for Protecting Travelers against Zoonotic Viruses

Anti-Gal activity varies in different individuals and at different ages [10,12,15,87]. Therefore, it is reasonable to assume that the efficacy of anti-Gal in mediating killing and neutralization of various zoonotic viruses presenting *α*-gal epitopes in the blood, GI tract, or airways may vary in different individuals. This raises the possibility that travelers to regions with high incidence of zoonotic virus infections may benefit from vaccination aimed to increase anti-Gal titers. Since many of the human B cells capable of producing anti-Gal are quiescent [14], immunization with *α*-gal epitopes linked to an immunogenic protein results in activation of the many anti-Gal B cells and thus in rapid elevation in anti-Gal titer. Such immunization of baboons with 1 mg of *α*-gal linked to bovine serum albumin resulted in increase of anti-Gal titer by as much as 200 folds [88]. A similar marked increase in anti-Gal titers was observed in patients receiving mouse cells [89] or pig cells [90] (i.e., cells presenting multiple *α*-gal epitopes) during experimental treatment with experimental therapies. An analogous example of increased protection by anti-Gal against pathogens presenting *α*-gal epitopes has been reported in studies on *Trypanosoma cruzi*, a protozoan pathogen presenting *α*-gal epitopes [91]. Immunization of a human volunteer with killed *Serratia marcescens* (an anti-Gal binding bacterium) [16] resulted in elevation in anti-Gal titer to a level that displayed effective complement mediated cytolysis of *T. cruzi* trypomastigotes [91,92]. A second example is that of anti-Gal interaction with *Plasmodium falciparum.* This malaria parasite was shown to present *α*-gal-like epitopes which readily bind anti-Gal [93,94,95,96,97]. Accordingly, these studies demonstrated significant elevation in anti-Gal activity in many subjects living in malaria endemic areas and in patients with acute *P. falciparum* malaria, in comparison with subjects living in areas where the incidence of *P. falciparum* malaria was scarce. Moreover, anti-Gal-mediated prevention of *P. falciparum* infection by the *Anopheles* mosquito was observed in anti-Gal producing GT-KO mice, whereas GT-KO mice lacking anti-Gal succumbed to the infection and developed malaria [96]. These studies with protozoan pathogens suggest that increased activity of anti-Gal by an *α*-gal vaccine may have similar protective effects in humans infected by zoonotic enveloped viruses presenting *α*-gal epitopes. Vaccination for elevating anti-Gal titers may be safe. This can be inferred from studies demonstrating that administration of porcine cells covered with glycoproteins presenting *α*-gal epitopes [98], or of glycolipids with linked *α*-gal epitopes [99], is safe in humans. In addition, production in humans of anti-Gal at high titers for prolonged periods was found to have no deleterious effects. This has been shown in diabetic patients transplanted with fetal porcine islet cells [90,98] and in patients implanted with porcine heart valves [100,101] or porcine tendon [102]. Elevated anti-Gal titers are usually kept in humans for a period of 2–4 months following the elimination of the immunizing *α*-gal epitopes [89,102]. Thus, the vaccination may be repeated if prolonged periods of high anti-Gal titers are required. Of particular interest would be a study on the effects of elevated anti-Gal titers on infection by Ebola virus since there are multiple glycans on this virus envelope glycoproteins. The structure of these glycans was found to be determined by the host-cells [103]. Thus, it is possible that Ebola virus produced in bats presents *α*-gal epitopes since bats are believed to be the natural reservoir of the virus, and bat cells display effective *α*-gal epitope synthesis [25]. It is of note that anti-Gal IgE mediates allergic reaction to meat in a small proportion of the population (referred to as *α*-gal syndrome) [104,105]. It is not clear whether intradermal or intramuscular immunization of such individuals has any adverse effects. The risk of such an allergic response upon administration of *α*-gal vaccine may be evaluated, and eliminated if needed by performing a skin test with the vaccine prior to the actual immunization.

## 7. Anti-Gal Associated Prevention of Extinction among Ancestral Old-World Monkeys and Apes

The ubiquitous synthesis of *α*-gal epitopes in non-primate mammals and not in other non-mammalian vertebrates implies that the *α*1,3GT enzyme synthesizing these epitopes appeared early in mammalian evolution (i.e., before the marsupial and placental mammals diverged from a common ancestor) [25]. The synthesis of *α*-gal epitopes in all New-World monkeys tested [24,25] strongly suggests that *α*1,3GT was active in ancestral Old-World primates, as in non-primate mammals, following the divergence from ancestral New-World monkeys, estimated to have occurred ~30–43 million years ago (mya) [106,107]. It is impossible, however, to determine the exact cause and period for the evolutionary conversion of ancestral Old-World monkeys and apes from *α*-gal epitope synthesizing primates to primates lacking the *α*-gal epitope and producing the natural anti-Gal antibody. Studies on the *α1,3GT* gene (*GGTA1*) and its pseudogene in Old-World primates (monkeys and apes) have provided a plausible scenario and estimated period for this evolutionary event in which anti-Gal protection against deadly viruses could prevent the complete extinction of ancestral Old-World primates. The *α1,3GT* gene originally cloned from murine [108] and bovine cDNA [109] was found to also be active in New-World monkeys [24,25,110]. However, this gene is present in Old-World monkeys, apes, and humans as a pseudogene inactivated by very few single base deletions generating premature stop codons that prevent production of catalytically active enzyme [27,28,29,30,110]. Apes (chimpanzees, gorillas, and orangutans) and humans have one conserved base deletion mutation (G904 in exon IX of the *α1,3GT* gene mouse sequence) [27,28,108]. Old-World monkeys (*rhesus macaque*) lack this deletion but have another base deletion in exon VII, which is also found in humans and orangutans [29]. Thus, it is not clear yet whether the accidental inactivation of the *α*1,3GT gene occurred before or shortly after the divergence between ancestral apes and monkeys [106,111] and is estimated to have occurred 20–30 mya. The absence of polymorphism in the inactivating mutations of the *α1,3GT* pseudogene in apes and humans suggests that this evolutionary event did not occur independently in a number of ancestral primate populations in different geographic regions of Eurasia–Africa (i.e., the “Old-World”), but rather in one small population of ancestral Old-World primates or in one population of ancestral Old-World monkeys and the other of ancestral apes.

**Figure 3:**
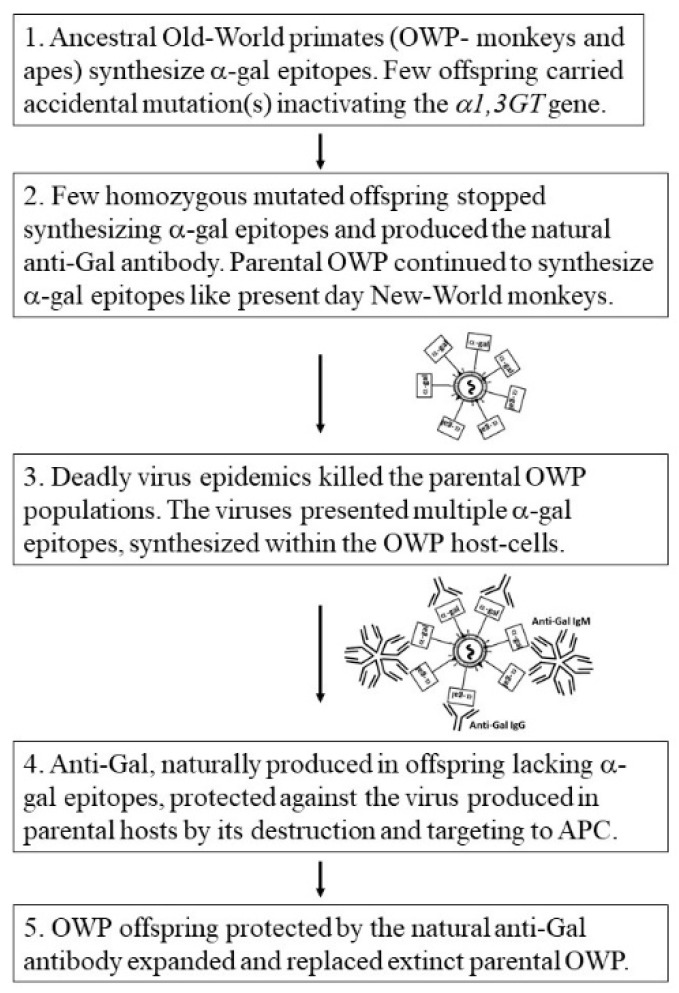
Hypothesis on the evolutionary stages which led to extinction of parental Old-World monkey and ape populations synthesizing *α*-gal epitopes and their replacement with offspring that lacked these epitopes because of mutational inactivation of the *α1,3GT* gene (*GGTA1*). These offspring produced the natural anti-Gal antibody that killed and neutralized the deadly viruses presenting *α*-gal epitopes (*α*-gal in rectangles) which were produced in parental populations, thereby enabling survival of Old-World primates (OWP).

The absence of Old-World monkeys and apes that synthesize *α*-gal epitopes [24,25] further implies that all parental Old-World primates synthesizing this epitope underwent extinction and only those who accidentally lost the *α*-gal epitope survived this selection process and expanded to become present day Old-World monkeys, apes, and humans. This evolutionary selection process may belong to the category of “Catastrophic selection process”. This process was defined in plants evolution by H. Lewis as “…an entire population is eliminated except for one or more exceptionally adapted individuals… characterized by a deviant genome” [112]. It is suggested that the natural anti-Gal antibody appearing de-novo in the few mutated offspring lacking *α*-gal epitopes had a key role in preventing the complete extinction of Old-World primates. Hypothetically, this selection process could occur in five steps according to the following suggested scenario (summarized in Figure 3): (1) An accidental base deletion mutation in the *α1,3GT* gene occurred in one of the *α*-gal epitope synthesizing ancestral Old-World primates, resulting in inactivation of one of the two *α1,3GT* alleles. Since this mutation is recessive, the mutated individual continued to synthesize *α*-gal epitopes. (2) After few generations of heterozygote progeny for the mutated *α1,3GT* gene, few homozygote offspring emerged in the population having two mutated alleles. Since such homozygous individuals stopped synthesizing the *α*-gal epitope and ceased to be immunotolerant to it, they produced anti-Gal as part of the antibody response to the multiple carbohydrate-antigens on GI bacteria. (3) Epidemics of deadly enveloped viruses in the Eurasia–Africa landmass effectively spread among parental primate populations synthesizing *α*-gal epitopes and caused extinction of these primates. The virus produced in these parental hosts presented *α*-gal epitopes, synthesized on envelope glycoproteins by *α*1,3GT conserved in the host-cells. (4) Anti-Gal produced in the very small populations of offspring that were homozygous for the mutated α1,3GT gene protected these offspring against the virus produced in the parental population by binding to *α*-gal epitopes on the virus envelope glycoproteins and destruction of the virus. Anti-Gal further amplified the specific anti-virus immune response by effective targeting of virus glycoproteins to APC, as illustrated in Figure 2. (5) The protected offspring expanded, underwent speciation, and replaced the extinct parental populations throughout the Eurasia–Africa landmass. This complete elimination of ancestral Old-World primates synthesizing *α*-gal epitopes did not occur on the island of Madagascar and in the continent of South America because of oceanic barriers that prevented the spread of the epidemics beyond Eurasia–Africa. Therefore, lemurs and New-World monkeys have not been subjected to this selection process and, thus, continue to synthesize *α*-gal epitopes and lack the anti-Gal antibody.

**Figure 4:**
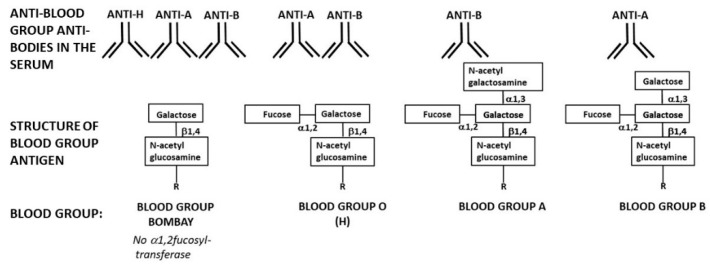
Schematic presentation of blood-group Bombay structure and the antibodies these individuals produce in comparison to the structure of blood-groups O (H), A, and B and the natural anti-ABO antibodies produced in individuals of these blood-groups. Note that only blood-group Bombay individuals naturally produce all three anti-H, anti-A, and anti-B antibodies.

There are several present day observations that support this suggested scenario: (1) The high efficacy of anti-Gal in human serum in inducing complement mediated destruction of viruses [66,67,68,69,70,71,72,73,74] further suggests that viruses produced in ancestral Old-World primates synthesizing *α*-gal epitopes were readily killed by this antibody in offspring lacking the *α*-gal epitopes and producing anti-Gal. The presence of maternal anti-Gal IgG in the human cord-blood [10] and of anti-Gal IgA in human colostrum and milk [11] suggests that anti-Gal protection against viruses presenting *α*-gal epitopes occurred also in newborns lacking *α*-gal epitopes. (2) The scenario above suggests that, once ancestral Old-World primate offspring became homozygous for the inactivated *α1,3GT* gene and stopped synthesizing *α*-gal epitopes, they immediately started producing the natural anti-Gal antibody because of elimination of immune tolerance to *α*-gal epitopes. This assumption is supported by the similar dynamics of immediate production of natural anti-Gal antibody upon elimination of *α*-gal epitopes, presently observed in pigs in which *α1,3GT* gene was inactivated by disruption (i.e., “knockout”) [113,114]. Whereas wild-type pigs lack anti-Gal, since they synthesize the *α*-gal epitope as an auto-antigen, *α1,3GT* knockout pigs (GT-KO pigs) lacking *α*-gal epitopes start producing the natural anti-Gal antibody at the age of six weeks and subsequently produce it at titers similar to those observed in humans [115,116,117]. (3) The proposed scenario assumes that among the large population(s) of ancestral Old-World primates producing *α*-gal epitopes there was a very small number of offspring homozygous for the mutation that inactivated the *α1,3GT* gene, resulting in lack of *α*-gal epitopes and production of anti-Gal in the mutated progeny. There is a present-day example among humans for similar inactivating mutations in a glycosyltransferase gene and the ensuing production of a natural antibody against the lost carbohydrate antigen. This is the rare blood-group “Bombay” individuals (called here “Bombay individuals”) who cannot synthesize blood-group O (also called the H-antigen) and produce natural anti-H antibody [118,119]. Bombay individuals lack the glycosyltransferase “*α*1,2fucosyltransferase” which in all other humans synthesizes the H-antigen (Figure 4) [9,119,120,121,122]. The prevalence of these individuals is between 1 in 10,000 in India and 1 in 1,000,000 in other countries [119,120,121,122,123]. Blood-group Bombay demonstrates an analogous stage to the pre-selection period in ancestral Old-World primates when a very small population of mutated primates lacked the *α*-gal epitope and produced the natural anti-Gal antibody. Blood-group Bombay is further discussed in a separate section below because of the hypothetical implications of the absence of the H-antigen and production of the natural anti-H antibody in future deadly virus epidemics.

## 8. Past Prevention of Hominin Extinction by the Natural Anti-Neu5Gc Antibody

The natural anti-Neu5Gc antibody, similar to anti-Gal, is produce in humans [44,45,46], and can mediate rejection of xenografts [45,47,49]. However, unlike anti-Gal, anti-Neu5Gc is absent in other Old-World monkeys and apes, all of which synthesize both Nue5Ac and Nue5Gc, as do most other non-primate mammalian species [39,40,41,124]. Therefore, it is reasonable to assume that most mammalian zoonotic viruses produced in Old-World monkeys, apes, and most non-primate mammalian reservoirs present Neu5Gc among the sialic acid units capping glycans on their envelope glycoproteins (Figure 1). Anti-Neu5Gc in human serum was found to induce destruction of viruses presenting Neu5Gc such as vesicular stomatitis virus (VSV) produced in Vero cells (African green monkey cells synthesizing Neu5Gc) by complement mediated lysis [62]. As with anti-Gal, there is no information on the extent of the anti-Neu5Gc contribution to actual prevention of infections by zoonotic viruses.

Synthesis of both Neu5Gc and Neu5Ac on glycans of chimpanzees, other apes, and Old-World monkeys vs. absence of Neu5Gc in humans strongly suggests that the catalytic activity for hydroxylation of Neu5Ac into Neu5Gc by CMAH was eliminated in hominins 2–6 mya [39,40,41,124]. Hominins are the ancestors of humans that lived following the evolutionary divergence from ancestors of chimpanzees (i.e., after the Homo–Pan divergence) that occurred ~6 mya. The inactivation of the *CMAH* gene by accidental deletion of a 92-bp region in this gene [39,42,43] was probably followed by production of the natural anti-Neu5Gc antibody in hominins homozygous for this mutation, analogous to production of anti-Gal in ancestral Old-World primates homozygous for the inactivated *α1,3GT* gene. This implies that the hydroxylation converting Neu5Ac into Neu5Gc turns this sialic acid into an antigen recognized by a natural antibody produced in humans, i.e., an antibody that can bind to foreign Neu5Gc, but not to self Neu5Ac.

Production of anti-Neu5Gc antibody further suggests that the extinction of hominins synthesizing both Neu5Ac and Neu5Gc occurred in association with a “catastrophic selection process” of a dynamics resembling that which replaced ancestral Old-World primates synthesizing *α*-gal epitopes with primates producing the natural anti-Gal antibody (Figure 3)**.** Since all apes synthesize both Neu5Ac and Neu5Gc, it is reasonable to assume that early hominins (after the split from ancestors of chimpanzees) also synthesized both Neu5Ac and Neu5Gc. It is suggested that a very small accidentally mutated population that lost the ability to synthesize Nue5Gc due to inactivation of the *CMAH* gene produced the natural anti-Neu5Gc antibody. In an epidemic of deadly enveloped virus(es) that killed parental hominin populations synthesizing both Neu5Gc and Neu5Ac, the natural anti-Neu5Gc antibody protected the few offspring lacking Neu5Gc and producing this antibody. This production of a natural antibody capable of binding to Neu5Gc but not to Neu5Ac has been conserved in humans that evolved from the selected hominins lacking Neu5Gc.

Evolutionary loss of Neu5Gc was reported also in several mammalian species including ferret, marten, weasel, hedgehog, white-tailed deer, sea lion, sperm whale, and New-World monkeys [124]. This suggests that the selection for survival of very small populations which accidentally lost Neu5Gc and produced the natural anti-Neu5Gc antibody has been an event that occurred in the course of evolution of various mammalian species in which this antibody could have protected from extinction by deadly viruses. This selection process has been feasible in these mammals probably because Neu5Ac is capable of fulfilling many of the biological functions of Neu5Gc in the absence of the latter sialic acid. Thus, similar to the *α*-gal epitope, Neu5Gc seems to be an expandable antigen in mammals.

## 9. The Natural Anti-Forssman Antibody and Mammalian Evolution

Analysis of glycolipids on the envelope of viruses produced in Forssman-antigen positive species (e.g., influenza virus grown in embryonated chicken eggs) demonstrated the prevalence of the Forssman-antigen glycolipid molecules which are included in the virus envelope in the process of budding from the membrane of host-cells [125]. Forssman-antigen was also detected on vaccinating pox virus [126]. The prevalent production of anti-Forssman antibody in humans [50,51,58] and the presence of Forssman glycolipid on viruses produced in Forssman-positive species raise the possibility that anti-Forssman antibody may have a role similar to that of anti-Gal and anti-Neu5Gc in protection against zoonotic viruses carrying the Forssman-antigen.

In addition to production of anti-Forssman antibody in humans, it was found in monkeys (baboon) and pigs [127]. The production of this antibody is likely to be the result of an immune response to Forssman-antigen shown to be present on bacteria of human flora [6,128]. Forssman-antigen is synthesized in only a number of mammalian species including sheep, dog, mouse, cat, horse, and guinea pig and is absent in rabbit, pig, cow, rat, monkeys, apes, and humans [55,56,57]. The demonstration of Forssman-antigen in non-mammalian vertebrates such as birds, amphibians, and fish [55,129,130,131] implies that it appeared early in vertebrate evolution. Furthermore, the glycosyltransferase gene coding Forssman synthetase [54] (also called *GBGT1* or *FS* gene) is present as a pseudogene, in humans in which there are two inactivating missense mutations [57]. Taken together, the activity of Forssman synthetase in non-mammalian vertebrates and its presence as a pseudogene in humans imply that this gene was inactivated in ancestors of the mammalian species lacking Forssman-antigen. The presence of Forssman-antigen on enveloped viruses produced in cells containing active Forssman synthetase suggests that the inactivation of this gene and the ensuing production of anti-Forssman antibody in humans, apes, and monkeys, as in other Forssman-negative mammals, could be associated with selection processes in the course of evolution of various mammalian lineages, similar to those described above for the *α*-gal epitope and the Neu5Gc antigen. In such an evolutionary scenario, catastrophic selection processes mediated by species-specific deadly virus epidemics, resulted in survival of mutated offspring in which the Forssman synthetase *GBGT1* gene was accidentally inactivated. Natural anti-Forssman antibody produced in these offspring protected the mutated offspring against the virus(es) that eliminated the Forssman positive parental populations. As with the *α*-gal epitope and Neu5Gc antigen, Forssman-antigen was expandable in the course of evolution of various mammalian species, thereby enabling survival of few accidentally mutated offspring lacking Forssman synthetase and producing natural anti-Forssman antibody.

## 10. The Natural Anti-H Antibody of Blood-Group Bombay in Future Viral Epidemics

A basic assumption in the hypothesis on the anti-MCA antibody mediated survival of mutated offspring in catastrophic selection processes is that the number of these mutated offspring is very small in comparison to the size of parental non-mutated populations. The surviving offspring lack an MCA common to the parental populations and produce a natural anti-MCA antibody against the lost MCA. As mentioned above, a present-day unique example of such a rare mutation in humans is blood-group Bombay individuals (referred to as Bombay individuals) with prevalence of 1 in 10,000 in India, 1 in 300,000 in Japan, and 1 in 1,000,000 in other countries [118,119,120,121,122,123]. The highest prevalence of one in several hundred was found on Réunion Island among descendants of 19th century migrators from India [120,121,122]. Bombay individuals cannot synthesize the H-antigen because they lack the H-transferase (Figure 4) [9,121,122]. The *FUT1* gene coding the glycosylation enzyme *α*1,2fucosyltransferase (*α*1,2FT) [132] displays in different ethnic groups of Bombay individuals a variety of missense and nonsense point mutations inactivating this gene [133,134,135,136,137,138]. In the rest of humans, *α*1,2FT synthesizes the H-antigen on glycans of RBC and nucleated cells (Figure 4). The polymorphism of these FUT1 gene inactivating mutations in different ethnic groups and the extreme rarity of Bombay individuals in indigenous African populations suggest that most of these accidental mutations appeared after humans migrated out of Africa to various geographical regions.

In the absence of the H-antigen, Bombay individuals produce the natural anti-H (anti-blood-group O) antibody [118,119,139] in a manner similar to the production of anti-Gal antibody, as described above. Since blood-groups A and B are synthesized on blood-group O (the H-carbohydrate antigen) (Figure 4), Bombay individuals cannot synthesize blood-group A or B antigens. Instead, they produce natural anti-blood-group A and anti-blood-group B antibodies, in addition to the production of anti-H antibody [118,119,140]. The potency of the anti-H antibody as an antibody inducing complement mediated activation and cytolysis has been demonstrated in a number of mismatched transfusions of O-type RBC to Bombay individuals, mistakenly typed as blood-group O. Such transfusions result in in-vivo complement mediated hemolysis of the transfused RBC by the natural anti-H antibody and may be lethal if not stopped in time and the patient treated [139,140,141].

Since humans of all ABO blood-groups, other than Bombay individuals, have active *α*1,2FT, some of the glycans they synthesize are capped with the H-antigen on glycolipids and glycoproteins. In blood-type A and B individuals, a proportion of the H-antigen epitopes is further converted into blood-group A or B antigens by linking GalNAc*α*1-3 or Gal*α*1-3, respectively, to the penultimate galactose of the glycan (Figure 4). Therefore, viruses produced in blood-group O human cells present the H-antigen on their envelope glycoproteins and viruses produced in blood-group A or B cells present the corresponding A- or B-antigen, in addition to the H-antigen [72,142,143]. Human sera containing anti-blood group A or B antibodies were shown to kill viruses expressing the corresponding blood-group antigen on their envelope [72,143]. In view of these studies, it may be assumed that the natural anti-A, -B, and -H antibodies produced in Bombay individuals are likely to destroy infecting enveloped viruses that were produced in any human host who is not of blood-group Bombay, since such viruses carry ABH antigens according to the host blood-type. The destruction of ABH carrying virus by the corresponding natural antibodies produced in Bombay individuals may occur by the same mechanism as that of anti-H mediated lysis of blood-group O RBC.

The production of anti-A, -B, and -H antibodies in Bombay individuals raises a hypothetical possibility that these antibodies may be of significance in future deadly enveloped virus epidemics, analogous to the significance of anti-Gal in ancestral Old-World monkeys and apes and that of anti-Neu5Gc in hominins. A timely production of effective vaccines against such a putative deadly virus may prevent epidemics in future vaccinated populations. However, in non-vaccinated populations, Bombay individuals may be immunoprotected by the natural anti-A, -B, and -H antibodies constantly produced in them, because enveloped viruses produced in human hosts that are not of blood group Bombay will carry ABH antigens on envelope glycans. One example for the anti-viral protective activity of anti-A and -B antibodies in humans is that mentioned above [75] on infection of healthcare workers who were not sufficiently protected while treating a patient with SARS-CoV infection in Hong Kong during the SARS epidemic of 2002–2003. Most of the infected workers were of blood-groups A, B, and AB, whereas blood-group O individuals were relatively resistant to this infection. Similarly, higher infection rates by influenza virus in annual influenza outbreaks were observed in blood-group AB individuals than of blood-group O individuals [144,145]. The lower infection rate in blood-group O individuals may be explained by their production of both anti-blood-group A and B antibodies, which destroy viruses produced in blood-groups A, B, and AB hosts. Such a protective effect may be further amplified in Bombay individuals because of the additional production of natural anti-H antibodies.

## 11. Conclusions

Human antibodies to Mammalian Carbohydrate-Antigens (MCA) are natural antibodies continuously produced against bacterial carbohydrate-antigens and binding to carbohydrate-antigens in mammals other than humans. Three of the important anti-MCA antibodies are anti-Gal, anti-Neu5Gc, and anti-Forssman antibodies binding to the ligands *α*-gal epitope, Neu5Gc, and Forssman-antigen, respectively. Since glycans on enveloped viruses produced in non-human mammalian cells are synthesized by the host glycosylation machinery, these viruses present on their envelope glycans at least one of these MCA. In-vitro studies demonstrating complement mediated killing of viruses by anti-MCA antibodies in human serum suggest that in-vivo binding of anti-MCA antibodies to MCA on zoonotic viruses results in similar destruction of such invading viruses. It is therefore suggested that anti-MCA antibodies serve as a first line of defense against zoonotic virus infections. It is further suggested that, in earlier evolutionary periods, accidental mutations resulting in elimination of MCA and production of the corresponding anti-MCA antibody prevented the extinction of the few mutated individuals in the course of deadly viral epidemics.

The *α*-gal epitope is synthesized in non-primate mammals, lemurs, and New-World monkeys. Accordingly, zoonotic enveloped viruses produced in these mammals present multiple *α*-gal epitopes on their glycans. This epitope is absence in Old-World monkeys, apes, and humans, all of which produce anti-Gal antibody in large amounts. Binding of anti-Gal in human serum to *α*-gal epitopes on viruses induces complement mediated killing and neutralization of viruses. In view of this virolytic activity of anti-Gal, it is suggested that this antibody may protect against zoonotic viruses produced in mammals that synthesize *α*-gal epitopes. Studies of the *α1,3GT* gene and pseudogene in primates imply that this gene was inactivated in ancestral Old-World primates after they split from New-World monkeys. Analysis of the *α1,3GT* pseudogene in apes and humans demonstrate a conserved single base deletion, which suggests the occurrence of a “catastrophic selection process” ~20–30 mya mediated by epidemics of deadly enveloped viruses. The viruses killed parental primates synthesizing *α*-gal epitopes, whereas few primates carrying homozygous accidentally mutated inactivated *α1,3GT* gene were protected by the natural anti-Gal antibody they produced and, thus, survived the epidemics and replaced the extinct parental populations.

Neu5Ac and Neu5Gc are synthesized in Old-World monkeys and apes, as well as in many non-primate mammals. The absence of Neu5Gc in humans implies that the gene coding the CMAH enzyme hydroxylizing Neu5Ac into Neu5Gc was accidentally inactivated in hominins after the split from ancestors of chimpanzee, 2–6 mya. The observed production of natural anti-Neu5c antibody in humans suggests that, similar to the protective activity of anti-Gal, anti-Neu5Gc could protect against zoonotic viruses presenting Neu5Gc. It is further suggested that anti-Neu5c antibody protected the few hominin offspring, accidentally lacking Neu5Gc, against deadly viral epidemics that eliminated parental hominins synthesizing Neu5Gc. The absence of Neu5Gc in various mammals (e.g., ferret, sperm whale, seal, and New-World monkeys) further suggests that the accidental loss of Neu5Gc and the ensuing production of the natural anti-Neu5Gc antibody have mediated a selection process that contributed to prevention from extinction of a number of mammalian species.

The anti-Forssman antibody is produced in humans against a glycolipid that is synthesized in a variety of mammalian species of different lineages and in non-mammalian vertebrates. Detection of this antigen on the envelope of viruses budding from host-cells synthesizing this antigen suggests that anti-Forssman antibody may contribute to protection against zoonotic viruses. Accidental mutations that led to elimination of Forssman-antigen in few offspring in species synthesizing this antigen and the ensuing production of the natural anti-Forssman antibody could result in survival of these offspring in viral epidemics and evolutionary conversion of a species from Forssman positive to Forssman negative phenotype, analogous to conversion of ancestral Old World primates from *α*-gal positive to *α*-gal negative phenotype and of ancestral hominins from Neu5Gc positive to Neu5Gc negative phenotype.

The scenario of accidental inactivation of a glycosyltransferase gene, loss of the corresponding carbohydrate antigen, and the subsequent production of a natural antibody against the lost carbohydrate antigen is demonstrated in the present-day example of blood-group Bombay individuals who comprise <0.001% of humans. These individuals lack the ability to synthesize the H-antigen (blood-group O-antigen) because of accidental mutations inactivating the H-transferase gene *FUT1* and produce natural anti-H antibody against the lost H-antigen. It is hypothesized that the natural anti-H antibody, in addition to anti-blood group A and B antibodies produced in blood-group Bombay individuals, may protect them against future lethal viral epidemics, because any enveloped virus produced in most humans (non-Bombay individuals) will carry at least one of the ABH antigens. Accordingly, the anti-MCA antibodies may play an important protective role against future infections by zoonotic viruses presenting *α*-gal epitopes, Neu5Gc, or Forssman-antigen. These considerations raise the possibility that vaccination for increasing anti-MCA antibody activity may increase immune protection of travelers to regions with many zoonotic viruses and other pathogens expressing ligands that bind the anti-MCA antibodies.

## Figures and Tables

**Table 1:** Major human natural antibodies binding to Mammalian Carbohydrate Antigens (MCA).

Natural Ab	Carbohydrate Ag	Species Producing Ab	Species Synthesizing Ag
Anti-Gal	Gal*α*1-3Gal*β*1-4GlcNAc-R (*α-gal epitope*)	Humans, apes, Old-World monkeys	Non-primate mammals, lemurs, New-World monkeys
Anti-Neu5Gc	Neu5Gc-R	Humans ^1^	Apes, Old-World monkeys, most non-primate mammals
Anti-Forssman	GalNAc*α*1-3GalNAc-R (*Forssman antigen*)	Humans, monkeys, pig	Sheep, horse, dog, cat, mouse, hamster, guinea-pig, non-mammalian vertebrates

^1^ No sufficient information about antibody activity in other species.
